# Clinical Outcomes and Applicability of Emergency Department Termination-of-Resuscitation Rules in Super-Elderly Patients with Out-of-Hospital Cardiac Arrest: A Multicenter Analysis

**DOI:** 10.3390/diagnostics16111653

**Published:** 2026-05-27

**Authors:** Yongkeun Park, Yujin Lee, Jeseop Lee, Tae-Youn Kim

**Affiliations:** Department of Emergency Medicine, Inha University Hospital, Inha University College of Medicine, Incheon 22212, Republic of Korea; pyk212@naver.com (Y.P.); eyeblack99@gmail.com (Y.L.); w154759@gmail.com (J.L.)

**Keywords:** emergency department, super-elderly, neurological outcome, prognosis, multicenter study

## Abstract

**Introduction**: The global aging population has led to a rapid increase in patients aged ≥ 80 years experiencing out-of-hospital cardiac arrest (OHCA). This study used data from three domestic university hospitals to analyze the clinical characteristics and prognoses of elderly patients and evaluate the validity of age-based criteria for termination of resuscitation (TOR). **Methods**: This study included 1234 adult patients with nontraumatic OHCA who presented to the emergency departments of three hospitals between 2015 and 2021. The patients were categorized as non-elderly (<65 years), elderly (65–79 years), or super-elderly (≥80 years), and outcomes, including return of spontaneous circulation (ROSC), survival to discharge, and favorable neurological outcomes (Cerebral Performance Category 1–2), were analyzed. Sensitivity, specificity, positive predictive value (PPV), negative predictive value (NPV) with Wilson 95% confidence intervals (CIs), and the area under the ROC curve (AUC) of the W−/D−/R− (unwitnessed/non-shockable/no prehospital ROSC) rule were calculated for both the full cohort and the super-elderly subgroup. **Results**: The super-elderly patients (*n* = 466) had significantly lower rates of ROSC, survival to discharge, and favorable neurological outcomes than the other age groups. Multivariate analysis revealed that extreme old age was a strong negative predictor of favorable neurological outcomes. For super-elderly patients as a whole (*n* = 466), the survival rate was only 2.4%, and the favorable neurological outcome rate was only 0.6%. **Conclusions**: Although the prognosis for super-elderly patients with OHCA is extremely poor, the possibility of survival is not entirely “zero.” Therefore, applying a multifactorial ED TOR rule that comprehensively considers whether the arrest was witnessed, the initial rhythm, and whether on-scene ROSC occurred, rather than relying solely on age criteria, would be more rational and aid in ethical decision-making.

## 1. Introduction

As the global population ages at an accelerating pace, the proportion of elderly patients experiencing out-of-hospital cardiac arrest (OHCA) is also rapidly increasing. According to the World Health Organization, the global population aged ≥ 60 years is projected to exceed 2 billion by 2050, which will lead to a sharp increase in the number of elderly patients with cardiac arrest [1,2]. Particularly, super-elderly patients aged ≥ 80 years have significantly lower survival rates and neurological outcomes than other age groups following cardiac arrest owing to their high prevalence of underlying conditions and diminished physical reserve capacity [3]. The overall survival rate for OHCA is approximately 8–12%; however, in the super-elderly patient population, it is significantly lower, at 2–5% [4,5]. Reviewing prior studies on the prognosis of OHCA in elderly patients, a study by Libungan et al. showed that the 30-day survival rate sharply decreased with increasing age [6]. A study by Okabayashi et al. analyzed 233,511 patients with OHCA aged ≥65 years and confirmed differences in prognosis based on location. For cardiac arrests in nursing homes, the rate of favorable neurological outcomes is only 0.6% [7]. Hiemstra et al. analyzed the long-term prognosis of elderly survivors using Dutch data. They reported that hospital survival rates were significantly lower in the elderly group than in the younger age group; however, approximately 73% of elderly survivors discharged from the hospital maintained a favorable neurological status of Cerebral Performance Category (CPC) 1–2 [8]. This suggests that even in elderly patients, a favorable prognosis can be expected when initial resuscitation is successful. However, these studies primarily target the entire elderly population aged ≥ 65 years or are based on Western data, leaving a lack of data specific to Korea’s medical environment and the super-elderly patient group aged ≥ 80 years [9,10].

In particular, recent advances in advanced critical care treatments, including extracorporeal cardiopulmonary resuscitation (ECPR) and targeted temperature management (TTM), have contributed to improved outcomes for patients with cardiac arrest [11,12]. There is debate over whether these advanced treatments yield the same efficacy in super-elderly patients. In the INCEPTION randomized controlled trial by Suverein et al., ECPR failed to demonstrate improved survival rates compared with conventional CPR [11], highlighting the need for further studies on the criteria for selective application in elderly patients. In Nielsen et al.’s TTM trial, there was no difference between managing body temperature at 33 °C and 36 °C [13]; however, subgroup analyses examining the impact of such interventions on the super-elderly population are limited. Furthermore, recent meta-analyses have presented conflicting results regarding whether age should be included when applying the termination of resuscitation (TOR) rules, necessitating concrete evidence on how emergency department (ED) TOR rules should be applied to this population.

Prehospital (basic life support [BLS] TOR) rules have been developed and validated primarily in North America and Europe, incorporating factors such as no prehospital return of spontaneous circulation (ROSC), non-shockable rhythm, and unwitnessed cardiac arrest [14,15]. In a study published in the *New England Journal of Medicine* in 2006, Morrison et al. demonstrated that the BLS TOR rule reduced unnecessary transfers by approximately 62.6% while only increasing the probability of missing survivors by 0.5% [14]. Subsequent guidelines from the American Heart Association (AHA) and the European Resuscitation Council (ERC) recommend the conditional application of these TOR rules [8,16]. Additionally, Goto et al. developed the ED TOR rule for emergency physicians, providing criteria for identifying patients with a low likelihood of resuscitation, even after hospital arrival [15,17]. This rule incorporates three elements: absence of prehospital ROSC, non-shockable initial rhythm, and unwitnessed cardiac arrest. When all these conditions are met, the positive predictive value (PPV) for predicting 1-month mortality is >99% [15]. The International Liaison Committee on Resuscitation 2020 Consensus on Science and Treatment Recommendations also conditionally recommended the use of the TOR rule, with the Universal TOR (UTOR) rule being evaluated as demonstrating the highest performance to date [15]. However, in some Asian countries, including Korea, the discontinuation of on-scene resuscitation is limited for legal and cultural reasons, and most patients are transported to the emergency room to receive advanced life support (ALS). From an ethical perspective, the resuscitation of the super-elderly also presents dilemmas. The ethics section of the American Heart Association 2025 guidelines recommends the decision to terminate resuscitation (TOR) based on a comprehensive consideration of the patient’s values, absence of reversible causes, and prolonged unresponsiveness [16]. The ERC 2021 and 2025 Guidelines for Resuscitation also state that if a patient remains in persistent asystole for >20 min after ALS and there is no reversible cause, TOR may be considered [18,19]. However, discontinuing resuscitation based solely on age may be considered age discrimination [20], and favorable outcomes have been reported even in super-elderly patients, necessitating a cautious approach [21].

This study aimed to analyze the clinical characteristics and prognoses of patients aged ≥ 80 years compared with non-elderly and elderly patients using multicenter OHCA data from three university hospitals. Specifically, we sought to (i) examine whether applying age as an independent criterion in ED-TOR rules is valid and (ii) quantitatively evaluate the diagnostic performance of the multifactorial ED-TOR rule (witnessed, defibrillated, prehospital ROSC) as a prognostic tool in the super-elderly population. The results of this study are expected to contribute to more rational and ethical decisions regarding the TOR efforts for elderly patients with cardiac arrest in emergency medical settings.

## 2. Materials and Methods

### 2.1. Study Design and Setting

This retrospective multicenter observational study was conducted in the ED of two tertiary university hospitals and one general hospital between January 2015 and December 2021. This study was approved by the Institutional Review Boards (IRBs) of Wonju Severance Christian Hospital (IRB no. CR322004), Dongguk University Ilsan Hospital, Dongguk University (IRB no. DUIH 2022-02-027), and National Health Insurance Service Ilsan Hospital (IRB no. NHIMC 2022-03-030-001). The study protocol conformed to the ethical guidelines of the Declaration of Helsinki (1975) and its amendments. As this study involved retrospective and observational analyses, the requirement for informed consent was waived, and patient records and information were anonymized before analysis. Emergency medical technicians provide both basic and ALS for a minimum of 5 min at the scene. If the ROSC cannot be achieved, the patient is transported to the nearest ED. After achieving ROSC, the patient is referred to the nearest hospital for post-cardiac arrest care, including TTM. All three centers follow the Korean Association of Cardiopulmonary Resuscitation (KACPR) and AHA guidelines for advanced cardiac life support and post-resuscitation care. Post-ROSC management followed institutional protocols based on the 2015 and 2020 AHA post-cardiac-arrest care guidelines, including TTM (33–36 °C) in eligible patients. Prehospital parameters (witnessed arrest, bystander CPR, defibrillation/initial shockable rhythm, prehospital CPR duration, and prehospital ROSC) were extracted directly from the EMS prehospital care report, a nationally mandated and structurally identical documentation system used by all 119 emergency medical services across the Republic of Korea.

### 2.2. Participants

This was a multicenter retrospective observational cohort study of adult patients with nontraumatic OHCA who presented to the emergency medical centers of three university hospitals between January 2015 and December 2021. Initially, 1636 patients were included in this study. We excluded 402 patients for the following reasons: (i) refusal of targeted temperature management (TTM) by the patient or family after informed consent (a preference-based exclusion, not a treatment-related one); (ii) traumatic OHCA; (iii) age < 18 years; (iv) death declared in the field with no transport to the ED; and (v) incomplete core variables. Finally, 1234 patients were included in the analysis.

### 2.3. Study Variables

Data were collected from the patients’ medical records and the Utstein-format cardiac arrest registration system [22,23]. The collected variables included age, sex, underlying conditions (hypertension, diabetes), bystander presence, bystander CPR administration, initial electrocardiogram rhythm (defibrillation feasibility), prehospital and in-hospital resuscitation times, and blood tests to identify reversible causes during resuscitation.

The patients were classified into three groups based on age: non-elderly (<65 years), elderly (65–79 years), and super-elderly (≥80 years). The primary outcome variables were ROSC, survival to discharge, and favorable neurological outcomes at discharge. Neurological outcomes were assessed using the CPC scale. CPC 1 (normal) and CPC 2 (moderately impaired) were classified as favorable outcomes, whereas CPC 3–5 were classified as unfavorable outcomes.

### 2.4. Statistical Analyses

Continuous variables are expressed as means ± standard deviations and categorical variables as frequencies and percentages (%). To compare the characteristics among the three age groups, analysis of variance was used for continuous variables and the chi-squared test for categorical variables. To assess the independent effect of extremely old age on prognosis, multivariate logistic regression analysis was performed after adjusting for sex, bystander presence, bystander CPR, and defibrillation. Odds ratios (ORs) and 95% confidence intervals (CIs) were determined. *p*-values < 0.05 were considered statistically significant. Multicollinearity was assessed using variance inflation factors (VIFs) prior to all multivariable analyses (Supplementary Table S1).

Diagnostic performance of the W−/D−/R− rule was characterized by sensitivity, specificity, PPV, and NPV with Wilson-method 95% confidence intervals. A cumulative TOR-score (0–3 negative factors) was used to construct ROC curves and to estimate the area under the curve (AUC) in both the full cohort and the super-elderly subgroup. All analyses were performed using SPSS version 23 (IBM Corp., New York, NY, USA) and R statistical software (version 3.6.3; R Foundation for Statistical Computing, Vienna, Austria). Graph generation and statistical analyses were performed in the Google Colab (Python version 3.12.12)) environment using matplotlib and seaborn libraries for data visualization. The scipy (version 1.16.3) and statsmodels packages (version 0.14.6) in Python were used for statistical analyses.

## 3. Results

### 3.1. General Characteristics

Among the 1234 patients, 692 (56.1%) were male, and the overall mean age was 71.1 ± 15.6 years. Among all the patients, 446 (36.1%) achieved ROSC. Of these, 80 (6.5%) were discharged alive, and 42 (3.4%) had favorable neurological outcomes (Table 1). Compared with the non-ROSC group, the ROSC group had a lower mean age (68.8 years vs. 72.5 years, *p* < 0.05), a higher proportion of witnessed cardiac arrests (63.7% vs. 41.1%, *p* < 0.05), and shorter total CPR duration (35.3 min vs. 51.3 min, *p* < 0.05).

### 3.2. Comparison of Prognosis According to Age Group

**Figure 1:**
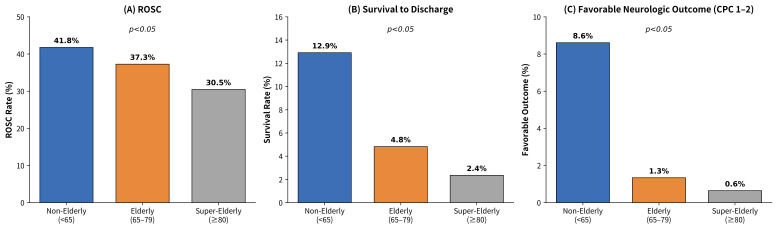
Comparison of prognosis according to age group: (**A**) Rate of return of spontaneous circulation (ROSC); (**B**) survival rate at discharge; (**C**) rate of favorable neurological outcomes (CPC 1–2). All outcomes showed a significant decrease with increasing age (*p* < 0.05). Bar graphs were generated using matplotlib in Google Colab (Python), and the chi-squared test was applied for intergroup comparisons.

Analysis dividing patients into age groups showed a decreasing trend in all survival indicators in the order of the non-elderly group (<65 years, *n* = 395), elderly group (65–79 years, *n* = 373), and super-elderly group (≥80 years, *n* = 466). The ROSC rate significantly decreased from 41.8% in the non-elderly group to 30.5% in the super-elderly group (*p* < 0.05), and the survival discharge rate also sharply decreased from 12.9% to 2.4% (*p* < 0.05). Notably, the clinically important favorable neurological outcome (CPC 1–2) was observed in 8.6% of the non-elderly group, 1.3% of the elderly group, and 0.6% of the super-elderly group, confirming the extremely low likelihood of neurological recovery in patients aged ≥ 80 years (Table 2) (Figure 1).

### 3.3. Multivariate Analysis of Prognostic Predictors

Multivariate logistic regression analysis for ROSC (Table 3) showed that the probability of achieving ROSC was significantly lower in the super-elderly (≥80 years) compared with the non-elderly group (OR, 0.65; 95% CI, 0.48–0.88; *p* < 0.001). Witnessed cardiac arrest was a strong positive predictor of ROSC (OR, 2.53; 95% CI, 1.97–3.25; *p* < 0.001).

Regarding survival at discharge (Table 4), the elderly (65–79 years) and the super-elderly (≥80 years) groups showed significantly lower survival rates compared with the non-elderly group (elderly group: OR, 0.38; 95% CI, 0.21–0.70; *p* = 0.002; the super-elderly group: OR, 0.16; 95% CI, 0.08–0.34; *p* < 0.001). Witnessed cardiac arrest (OR, 1.88; 95% CI, 1.08–3.29; *p* = 0.027) and shockable rhythm (OR, 2.10; 95% CI, 1.20–3.70; *p* = 0.010) were significant positive predictors of survival. Regarding favorable neurological outcomes (Table 5), the super-elderly group was a very strong negative predictor compared with the non-elderly group (OR, 0.05; 95% CI, 0.01–0.22; *p* < 0.001). Shockable rhythm was the strongest positive predictor of favorable neurological outcomes (OR, 4.41; 95% CI, 2.05–9.51; *p* < 0.001). This finding suggests that advanced age is a critical factor that limits neurological recovery beyond survival.

### 3.4. ED-TOR Rule Diagnostic Performance and W/D/R Combinations

**Figure 2:**
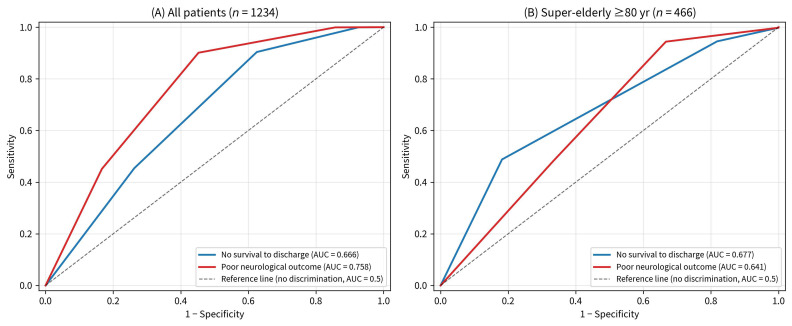
Receiver operating characteristic (ROC) curves of the cumulative ED termination-of-resuscitation (TOR) score for predicting no survival to discharge and poor neurological outcome.

A prognostic analysis was performed considering the combination of prehospital witnessed status (W), defibrillation/shockable rhythm (D), and prehospital ROSC (R). The diagnostic performance of the W−/D−/R− rule was calculated for both the full cohort and the super-elderly subgroup, with Wilson 95% CIs (Table 6). The cumulative TOR-score (0–3 negative factors) was used to construct ROC curves (Figure 2).

**Figure 3:**
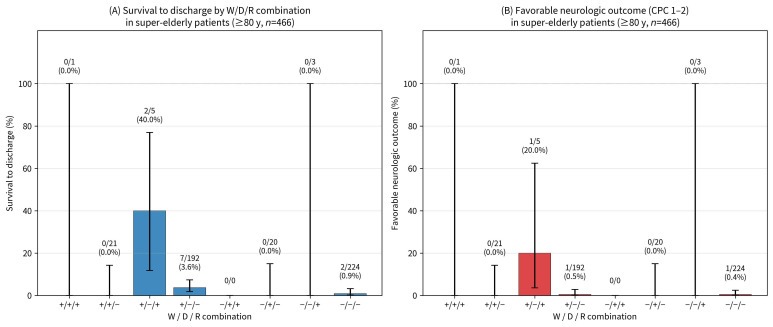
Prognosis by ED-TOR combination in the super-elderly group (≥80 years, *n* = 466): (**A**) survival to discharge; (**B**) favorable neurologic outcome.

Analysis of prognosis based on the combination of prehospital witnessed status (W), defibrillation/shockable rhythm (D), and prehospital ROSC (R) showed the best outcomes when all three factors were positive (W+/D+/R+) in the full cohort (survival 85.7% [6/7]; favorable neurologic outcome 85.7% [6/7]) (Table 7). Conversely, when all three factors were negative (W−/D−/R−), the survival rate was 3.9% (21/545; 95% CI 2.5–5.8) and the favorable neurologic outcome rate was 1.3% (7/545; 95% CI 0.6–2.6). In the super-elderly subgroup, however, the W+/D+/R+ combination contained only one patient (who did not survive), and the W+/D+/R− combination contained 21 patients with no survivors, so the apparent benefit of multiple favorable factors observed in the full cohort could not be confirmed in the super-elderly subgroup (Table 7, Figure 3).

Subgroup analysis within the super-elderly group revealed that among the 224 patients with the W−/D−/R− combination, only two (0.9%, 95% CI 0.2–3.2) survived and only one (0.4%, 95% CI 0.1–2.5) had a favorable neurologic outcome. Even in the W+/D−/R− combination (*n* = 192), the survival rate was only 3.6% (7/192; 95% CI 1.8–7.3), confirming that witnessed status alone cannot guarantee a favorable prognosis in this age group. Within the small super-elderly subgroups in which both witnessed arrest and shockable rhythm were present in the absence of prehospital ROSC, no survivor was observed (W+/D+/R+: 0/1, 95% CI 0.0–79.3; W+/D+/R−: 0/21, 95% CI 0.0–15.5), although the very small sample sizes preclude firm conclusions about these specific combinations. Of the 11 super-elderly survivors, 2 occurred in the W+/D−/R+ subgroup (40.0%, 95% CI 11.8–76.9)—the only combination with a meaningfully elevated survival probability in this cohort.

## 4. Discussion

This study analyzed the prognosis of super-elderly patients with OHCA using multicenter data from three university hospitals in Korea and examined the applicability of age-based ED TOR. Key findings showed that the neurological improvement discharge rate for patients aged ≥80 years was only 0.6% (3/466), significantly lower than that of the non-elderly group (8.6%). Furthermore, in multivariate analysis, super-elderly was a strong independent predictor of poor outcomes. Specifically, the probability of achieving a favorable neurological outcome in the super-elderly group was reduced by 95% compared with the non-elderly group, confirming that age has an extremely significant impact on neurological recovery. However, all of these very few survivors had favorable conditions. These results are consistent with the existing international studies reported by Hiemstra et al. [8] and Okabayashi et al. [7], reaffirming that increasing age reduces resuscitation responsiveness owing to diminished physiological reserve capacity and increased comorbidities. A study also reported a 30-day survival rate of 4.4% in patients aged 80–89 years, showing a pattern similar to the 2.4% observed in this study [6]. Funada et al. analyzed trends in OHCA prognosis improvement according to age group and reported that survival rates improved over time, even in the 75–84 and 85–94 age groups, although they remained significantly lower than those in younger age groups [24]. Nevertheless, the existence of a very small number of super-elderly survivors indicates that the premature discontinuation of resuscitation based solely on age carries the risk of excluding potentially viable patients. A notable finding in this study was the result of the multivariate analysis. Witnessed cardiac arrest was the strongest positive predictor for ROSC, consistent with previous Utstein-based studies [25,26]. In contrast, bystander CPR showed no significant effect in this study, which differs from the results of previous studies [27,28]. These differences may be attributed to variations in the quality of layperson CPR in Korea, differences in emergency medical service arrival times, and the limitations of the sample size of this study. Shockable rhythm was a strong positive predictor of all prognostic indicators. It showed the strongest effect, particularly for ROSC, survival to hospital discharge, and favorable neurological outcomes. This suggests that an initial shockable rhythm has a decisive impact not only on the simple ROSC but also on long-term neurological outcomes. CPR duration was consistently identified as a negative predictor of all outcomes, reaffirming the importance of early resuscitation [29,30].

Notably, despite the extremely poor prognosis for super-elderly patients, survival and neurological recovery are not “zero.” In this study, three of 466 patients aged ≥ 80 years (0.6%) were discharged with a CPC of 1–2. Although the absolute number was small, most of these cases involved a witnessed cardiac arrest with an initial rhythm amenable to defibrillation. Importantly, all 11 super-elderly survivors clustered in subgroups where either prehospital ROSC was achieved (W+/D−/R+: 2 survivors of 5; 40.0%, 95% CI 11.8–76.9) or where prehospital CPR ultimately succeeded despite an absence of all three favorable factors (W+/D−/R−: 7 of 192; 3.6%, 95% CI 1.8–7.3; W−/D−/R−: 2 of 224; 0.9%, 95% CI 0.2–3.2). In contrast, no survivor was observed in the small super-elderly subgroups with witnessed shockable arrest in the absence of prehospital ROSC (W+/D+/R+: 0/1; W+/D+/R−: 0/21), indicating that, in this age group, the presence of favorable initial factors alone does not guarantee survival when in-hospital resuscitation is required. This suggests that determining the cessation of resuscitation based solely on the single factor of age lacks ethical and medical validity [20,31]. In fact, a Danish study found that 70% of OHCA survivors aged ≥80 years maintained CPC 1–2 status 30 days later, showing no significant difference compared with younger age groups [21]. These results indicate that although the neurological prognosis for “successfully resuscitated” super-elderly patients may be favorable, the success rate of resuscitation itself is low. Recent meta-analyses also recommend applying validated TOR rules (e.g., UTOR or Goto ED TOR rules) that encompass on-site response time, presence of witnesses, and initial rhythm, rather than relying solely on age criteria [4]. The ED TOR rule developed by Goto et al. (no prehospital ROSC + unwitnessed + non-shockable rhythm) is capable of identifying patients with poor prognosis with high specificity (90.3%) and PPV (99.3%) [15]. Subsequently, Goto et al. proposed a modified rule incorporating prehospital CPR duration, which included cases in which EMS CPR exceeded 20 min. This rule is validated using nationwide data from Japan [17]. The results of the ED TOR combination analysis performed in this study confirmed the validity of these rules.

In our super-elderly cohort, 1 in 42 resuscitation attempts resulted in survival to discharge, but only 1 in 155 yielded a favorable neurologic outcome; within the W−/D−/R− super-elderly subgroup, only 1 in 224 attempts yielded a favorable neurologic outcome. These numbers must be interpreted in the context of finite ED resources, the documented higher healthcare cost per elderly OHCA patient [9], and the centrality of pre-arrest patient values, advance directives, and pre-arrest quality of life in shared decision-making. Accordingly, the multifactorial ED-TOR rule (W/D/R) should be regarded as a supportive tool that enables, rather than replaces, this nuanced clinical and ethical judgment, and age alone should not serve as the sole criterion for termination of resuscitation. Compared with many other countries, Korea more actively performs cardiopulmonary resuscitation in elderly and super-elderly patients, which gives the present cohort a distinctive value in characterizing outcomes in this age group. Nevertheless, decisions regarding termination of resuscitation must be considered within each country’s specific legal and cultural context.

However, this study has some limitations. First, as this was a retrospective observational study, the possibility of selection and information biases could not be excluded. In particular, because patients declared dead before hospital arrival or those with a DNR order were excluded from this study, the overall prognosis of the super-elderly patients with OHCA may be worse than the results of this study. Second, the study period (2015–2021) may not fully reflect recent advances in resuscitation techniques and post-resuscitation care (e.g., TTM, ECPR). This period was chosen to exclude the impact of coronavirus disease 2019-related difficulties in registry collection and the effects of CPR performed after this period. Third, as the data from a single region were limited to three university hospitals, generalizability to the entire population or other healthcare settings was constrained. Fourth, information related to the patients’ overall quality of life was insufficiently collected, and qualitative elements could not be analyzed. Fifth, the registry did not collect frailty indices, pre-arrest performance status, nursing-home residence, or other measures of functional baseline. Because these variables are known to substantially influence prognosis in super-elderly patients, their absence is an important limitation. Sixth, because only three super-elderly patients achieved a favorable neurologic outcome (events = 3 per 466 patients), the subgroup-level multivariable estimates have very wide confidence intervals and are statistically unstable. These estimates should therefore be interpreted as hypothesis-generating rather than as definitive effect estimates (Supplementary Table S2).

## 5. Conclusions

In this multicenter cohort of 1234 OHCA patients, super-elderly patients (≥80 years) had survival to discharge of 2.4% and a favorable neurologic outcome rate of 0.6%. In this subgroup, the W−/D−/R− criterion achieved a positive predictive value of 99.1% for no survival to discharge and 99.6% for poor neurologic outcome, while misclassifying 18.2% of survivors and 33.3% of patients with favorable neurologic outcomes. These findings support a multifactorial ED-TOR rule, rather than age alone, as a decision-support adjunct, although prospective external validation is warranted before clinical implementation.

## Figures and Tables

**Table 1:** Baseline characteristics of the study patients.

Characteristics	All Patients (*n* = 1234)	Non-ROSC (*n* = 788)	ROSC (*n* = 446)	*p*-Value
Age (years)	71.1 ± 15.6	72.5 ± 15.5	68.8 ± 15.5	<0.05
Sex (male)	692 (56.1%)	436 (55.3%)	256 (57.4%)	0.520
Hypertension, n (%)	547 (44.3)	353 (44.8)	194 (43.5)	0.703
Diabetes, n (%)	363 (29.4)	221 (28.0)	142 (31.8)	0.180
Witnessed arrest	608 (49.3%)	324 (41.1%)	284 (63.7%)	<0.05
Bystander CPR	682 (55.3%)	428 (54.3%)	254 (57.0%)	0.404
Defibrillation	207 (16.8%)	112 (14.2%)	95 (21.3%)	<0.05
Prehospital ROSC, n (%)	22 (1.8)	6 (0.8)	16 (3.6)	<0.001
Prehospital CPR (min)	25.9 ± 16.7	28.6 ± 17.5	21.2 ± 14.2	<0.001
In-hospital CPR (min)	19.5 ± 14.1	22.6 ± 13.3	14.1 ± 13.8	<0.001
Total CPR time (min)	45.5 ± 22.4	51.3 ± 21.5	35.3 ± 20.2	<0.05

**Table 2:** Clinical outcomes stratified according to age group.

Outcome	Non-Elderly (<65) (*n* = 395)	Elderly (65–79) (*n* = 373)	Super-Elderly (≥80) (*n* = 466)	*p*-Value
ROSC	165 (41.8%)	139 (37.3%)	142 (30.5%)	<0.05
Survival to discharge	51 (12.9%)	18 (4.8%)	11 (2.4%)	<0.05
Favorable neurological outcome (CPC 1–2)	34 (8.6%)	5 (1.3%)	3 (0.6%)	<0.05

**Table 3:** Multivariate logistic regression for factors related to ROSC.

Variable	Odds Ratio (OR)	95% Confidence Interval	*p*-Value
Age group			
Non-elderly (<65)	Reference		
Age 65–79 (vs. <65)	0.88	0.65–1.20	0.428
Age ≥ 80 (vs. <65)	0.65	0.48–0.88	0.005
Male sex	1.11	0.86–1.42	0.429
Witnessed arrest	2.53	1.97–3.25	<0.001
Bystander CPR	0.88	0.69–1.13	0.322
Shockable rhythm	1.33	0.96–1.83	0.087

**Table 4:** Multivariate logistic regression for survival to discharge.

Variable	Odds Ratio (OR)	95% Confidence Interval	*p*-Value
Age group			
Non-elderly (<65)	Reference		
Elderly (65–79)	0.38	0.21–0.70	0.002
Super-elderly (≥80)	0.16	0.08–0.34	<0.001
Sex (male)	0.86	0.50–1.47	0.580
Bystander CPR	1.07	0.62–1.82	0.816
Witnessed arrest	1.88	1.08–3.29	0.027
Defibrillation (shockable rhythm)	2.10	1.20–3.70	0.010
Total CPR time (min)	0.95	0.93–0.96	<0.001

**Table 5:** Multivariate logistic regression for favorable neurological outcomes.

Variable	Odds Ratio (OR)	95% Confidence Interval	*p*-Value
Age group			
Non-elderly (<65)	Reference		
Elderly (65–79)	0.15	0.05–0.44	<0.001
Super-elderly (≥80)	0.05	0.01–0.22	<0.001
Sex (male)	1.54	0.71–3.37	0.275
Bystander CPR	0.91	0.41–2.03	0.825
Witnessed arrest	2.73	1.11–6.73	0.029
Defibrillation (shockable rhythm)	4.41	2.05–9.51	<0.001
Total CPR time (min)	0.94	0.91–0.96	<0.001

**Table 6:** Diagnostic performance of the W−/D−/R− rule for predicting no-survival discharge and poor neurologic outcome.

Population/Outcome	Sensitivity (95% CI)	Specificity (95% CI)	PPV (95% CI)	NPV (95% CI)	AUC	Missed Survivors
All (*n* = 1234) no survival discharge	45.4% (42.6–48.3)	73.8% (63.2–82.1)	96.2% (94.2–97.5)	8.6% (6.7–10.9)	0.666	21/80 (26.3%)
All poor neurological outcome	45.1% (42.3–48.0)	83.3% (69.4–91.7)	98.7% (97.4–99.4)	5.1% (3.7–7.0)	0.758	7/42 (16.7%)
super-elderly (*n* = 466) no survival discharge	48.8% (44.2–53.4)	81.8% (52.3–94.9)	99.1% (96.8–99.8)	3.7% (2.0–6.9)	0.677	2/11 (18.2%)
Super-elderly poor neurological outcome	48.2% (43.6–52.7)	66.7% (20.8–93.9)	99.6% (97.5–99.9)	0.8% (0.2–3.0)	0.641	1/3 (33.3%)

PPV = positive predictive value; NPV = negative predictive value; AUC = area under the ROC curve.

**Table 7:** Outcomes by W/D/R combination, with Wilson 95% confidence intervals.

W/D/R	N	Survival to Discharge (95% CI)	Favorable Neurologic Outcome (95% CI)
All patients (*n* = 1234)			
+/+/+	7	85.7 (48.7–97.4)	85.7 (48.7–97.4)
+/+/−	124	15.3 (10.0–22.7)	10.5 (6.2–17.1)
+/−/+	10	50.0 (23.7–76.3)	40.0 (16.8–68.7)
+/−/−	467	5.6 (3.8–8.0)	2.1 (1.2–3.9)
−/+/−	76	3.9 (1.4–11.0)	2.6 (0.7–9.1)
−/−/+	5	0.0 (0.0–43.4)	0.0 (0.0–43.4)
−/−/−	545	3.9 (2.5–5.8)	1.3 (0.6–2.6)
Super-elderly ≥ 80 (*n* = 466)			
+/+/+	1	0.0 (0.0–79.3)	0.0 (0.0–79.3)
+/+/−	21	0.0 (0.0–15.5)	0.0 (0.0–15.5)
+/−/+	5	40.0 (11.8–76.9)	20.0 (3.6–62.4)
+/−/−	192	3.6 (1.8–7.3)	0.5 (0.1–2.9)
−/+/−	20	0.0 (0.0–16.1)	0.0 (0.0–16.1)
−/−/+	3	0.0 (0.0–56.2)	0.0 (0.0–56.2)
−/−/−	224	0.9 (0.2–3.2)	0.4 (0.1–2.5)

## Data Availability

The data presented in this study are available on request from the corresponding author due to patient consent for the use of personal information.

## Supplementary Materials

The following supporting information can be downloaded at: https://www.mdpi.com/article/10.3390/diagnostics16111653/s1, Supplementary Table S1: Variance inflation factors (VIF) for predictors in the primary multivariable models. All VIFs < 2.5, indicating no problematic multicollinearity. Supplementary Table S2: Firth’s penalized logistic regression in the super-elderly subgroup (*n* = 466). Used to address sparse-events bias and complete separation observed with standard maximum-likelihood estimation. Pre-specified predictors: sex, witnessed, bystander CPR, shockable rhythm.
