# Supplementary material for: Clinical Outcomes and Applicability of Emergency Department Termination-of-Resuscitation Rules in Super-Elderly Patients with Out-of-Hospital Cardiac Arrest: A Multicenter Analysis
Source: Diagnostics (Basel). 2026 May 27;16(11):1653. doi: 10.3390/diagnostics16111653 (PMC13256758; doi:10.3390/diagnostics16111653)
Supplement: Supplementary file 1 [file diagnostics-16-01653-s001.zip › diagnostics-4306598-supplementary.pdf]

Supplementary Table S1. Variance inflation factors (VIF) for predictors in the primary multivariable models. All VIFs <2.5, indicating no problematic multicollinearity.

Predictor	VIF
Male sex	1.72
Witnessed arrest	1.95
Bystander CPR	2.17
Shockable rhythm	1.22
Age 65–79	1.62
Age ⩾80	1.86
Prehospital CPR duration	2.37

Supplementary Table S2.

Firth's penalized logistic regression in the super-elderly subgroup (n = 466). Used to address sparse-events bias and complete separation observed with standard maximum-likelihood estimation. Pre-specified predictors: sex, witnessed, bystander CPR, shockable rhythm.

Outcome	Predictor	OR (Firth)	95% CI	p
ROSC (events = 142)	Male sex	1.54	1.02–2.33	0.042
	Witnessed arrest	2.79	1.84–4.22	<0.001
	Bystander CPR	0.97	0.64–1.47	0.899
	Shockable rhythm	0.94	0.46–1.93	0.864
Survival (events = 11)	Male sex	0.69	0.23–2.08	0.509
	Witnessed arrest	4.13	1.08–15.77	0.038
	Bystander CPR	1.62	0.49–5.41	0.431
	Shockable rhythm	0.38	0.02–5.91	0.487
Favorable neurologic outcome (events = 3)	Male sex	0.49	0.08–2.88	0.428
	Witnessed arrest	1.77	0.31–10.27	0.525
	Bystander CPR	1.24	0.21–7.21	0.809
	Shockable rhythm	1.18	0.08–16.55	0.904

Firth's correction was applied to all super-elderly subgroup models because of sparse outcomes. For the favorable-neurologic outcome (events = 3), confidence intervals remain wide and the estimates should be interpreted as hypothesis-generating. For survival (events = 11), witnessed arrest remained significantly associated with the outcome.
